# Chemical Composition and Fatty Acid Content of Some Spices and Herbs under Saudi Arabia Conditions

**DOI:** 10.1100/2012/859892

**Published:** 2012-12-24

**Authors:** Fahad Mohammed Al-Jasass, Mohammed Saud Al-Jasser

**Affiliations:** ^1^General Directorate of Research Grants, King Abdulaziz City for Science and Technology, P.O. Box 6086, Riyadh 11442, Saudi Arabia; ^2^Department of Food Science and Nutrition, College of Food and Agricultural Sciences, King Saud University, P.O. Box 2460, Riyadh 11451, Saudi Arabia

## Abstract

Some Saudi herbs and spices were analyzed. The results indicated that mustard, black cumin, and cress seeds contain high amount of fat 38.45%, 31.95% and 23.19%, respectively, as compared to clove (16.63%), black pepper (5.34%) and fenugreek (4.51%) seeds. Cress, mustard, black cumin and black pepper contain higher protein contents ranging from 26.61 to 25.45%, as compared to fenugreek (12.91%) and clove (6.9%). Crude fiber and ash content ranged from 6.36 to 23.6% and from 3.57 to 7.1%, respectively. All seeds contain high levels of potassium (ranging from 383 to 823 mg/100g), followed by calcium (ranging from 75 to 270 mg/100g), Magnesium (ranged from 42 to 102 mg/100g) and iron (ranged from 20.5 to 65 mg/100g). However, zinc, manganese and copper were found at low levels. The major fatty acids in cress and mustard were linolenic acid (48.43%) and erucic acid (29.81%), respectively. The lenoleic acid was the major fatty acid in black cumin, fenugreek, black pepper and clove oils being 68.07%, 34.85%, 33.03% and 44.73%, respectively. Total unsaturated fatty acids were 83.24, 95.62, 86.46, 92.99, 81.34 and 87.82% for cress, mustard, black cumin, fenugreek, black pepper and clove, respectively. The differences in the results obtained are due to environmental factors, production areas, cultivars used to produce seeds and also due to the different methods used to prepare these local spices.

## 1. Introduction

Some seeds are grown primarily for their use as condiments or for herbal medicine such as fenugreek (*Trigonella foenum-gracecum* L.), cress (*Lepidium sativum* L.), mustard (*Sinapis alba* L.), black cumin (*Nigella sativa* L.), black pepper (*Piper nigrum* L.), and clove (*Dianthus *sp.). Fenugreek is an annual crop belonging to the Fabaceae family. This crop is native to an area extending from Iran to northern India, but is now widely cultivated in China, north and east Africa, Ukraine, and Greece [1]. In parts of Asia, the young plants are used as pot herbs and the seeds as a spice or as herbal medicine [2]. Medically, fenugreek is reported to have antidiabetic, antifertility, anticancer, antimicrobial, and antiparasitic, and hypocholesterolaemic effects [3]. Seeds contain 26% mucilage, 22% protein comprising of globulin, histidine, and albumin with a good amount of phosphorus, sulphur, and also lecithin, it contains also 50% of soluble and insoluble fiber found essential for good health [4]. Fenugreek oil contains *ω*-3, *ω*-6, and *ω*-9 fatty acids along with many saponins, alkaloids, and sterols that serve as a source of proestrogens and inhibit intestinal cholesterol absorption [5]. Kochhar et al. [6] reported that fenugreek seeds contain 11.8% moisture, 25.8% crude protein, 6.53% oil, 3.26% ash, and 6.28% crude fiber and 58.13% total carbohydrates on dry basis. However, El-Nasri and El-Tinay [7] found that protein content of fenugreek was found to be 28.4%, crude fiber content was 9.3%, and crude fat was 7.1%. The fatty acid profile was dominated by unsaturated acids, namely, oleic, linoleic, and linolenic acids accounting for 16.3%, 50% and 24.4%, respectively of the total fatty acids. However, El-Sebaiy and El-Mahdy [8] reported that the fatty acids C_18:2_ and C_18:3_ were the most abundant fatty acids in the lipids of the fenugreek seeds. Thus fenugreek seeds may serve to be a beneficial health food if consumed regularly.

Cress is known as garden cress or garden cress pepper weed, and it is a fast growing annual herb. It belongs to the Brassicaceae family that is native to Egypt and west Asia but is widely cultivated in temperate climates throughout the world for various culinary and medicinal uses [9]. It was also reported cress seeds contain 22.5% protein, 27.5% fat, 30% dietary fiber, and 1193 mg/100 g potassium. Hence, it was assumed that these seeds can be used as a functional food. Moreover, Moser et al. [10] found that the oil content of dried cress seeds was 22.7% and the primary fatty acids found in cress oil were oleic (30.6%) and linolenic acids (29.3%). Cress oil contained high concentrations of *γ*-(1422 ppm) and (356 ppm) tocopherols. However, Gokavi et al. [9] reported that the primary fatty acids found in cress oil were oleic (C_18:1_; 30.6%), linolenic (C_18:3_; 29.3%), palmitic (C_16:0_; 9.4%), linoleic (C_18:2_; 7.6%), erucic (C_22:1_; 3.0%), stearic (C_18:0_; 2.8%), and arachidic (C_20:0_; 2.3%) acids among the minor fatty acids found in cress oil.

Mustard is a herb belonging to the Brassicaceae family and the dry seeds are the only part used. It stimulates digestion and salivary secretion [11]. Mustard seeds have an advantageous chemical composition such as its protein content and fairly well-balanced amino acid composition, rich in dietary fiber and natural antioxidants. In addition to its nutritional value, mustard seed flour offers rather unique functional properties; therefore, it could be taken into consideration as potential component of many food products [12]. White mustard has been used effectively for food and medical applications, one of the limiting factors for human use of mustard products is the spicy flavor produced by myrosinase enzyme activities. Mustard seeds have high-energy content, having 28–32% oil with relatively high protein content (28–36%). The amino acid composition of mustard protein is well balanced; it is rich in essential amino acids. Mustard seeds until now have been used mainly for condiment production, however, this advantageous chemical composition and its relatively low price offer wide possibilities for utilization of this valuable seed, for example, in human foods as additive and to feed animals. Mustard oil has a special fatty acid composition, it contains about 20–28% oleic acid, 10–12% linoleic, 9.0–9.5% linolenic acid, and 30–40% erucic acid, which is indigestible for human and animal organisms. The high erucic acid content of mustard seed could be reduced by breeding, some low erucic acid content genotypes are in cultivation in several countries. Mustard oil is rich in tocopherols, as a consequence of their antioxidant characteristic, they act as a preservative against rancidity [10].

Black cumin is a herb belonging to the Ranunculaceae family and it is widely distributed in countries bordering the Mediterranean Sea, middle Europe, and western Asia [13]. The seeds of N. sativa have been known also as black cumin or black caraway in English and corek out in Turkish, and used as spice and culinary purposes [14]. Black cumin contains 30 to 40% oil and 20 to 30% protein, 3.7–4.7% ash and 25–40% total carbohydrates with antioxidants lignans such as saponin, melantin [15]. Fatty acid compositions of black cumin were C_14:0_ (12.97–13.23%), C_16:0_ (9.47–13.34%), C_18:1_ (15.17–24.15%), and C_18:2_ (54.32–70.81%) as reported by Cheikh-Rouhou et al. [13] and Tulukcu [16]. On the other hand, black cumin oil is considered as one among the newer sources of edible oils. Linoleic acid, undoubtedly one of the most important polyunsaturated fatty acids in human food because of its prevention of distinct heart vascular diseases is present in all the seed oils [17]. It was revealed that the oleic and linoleic acids are the most abundant monounsaturated and polyunsaturated fatty acids in all samples, respectively. The total MUFA composition of the studied species is assigned between 15.17 and 24.15%.

Pepper is a flowering vine belonging to the Piperaceae family and is the most widely used of all condiments. The components contributing to its value are the alkaloids, of which piperine is the most important, for pregnancy, and the volatile (essential) oil for odor and flavor as well as for massage [18]. Black pepper contains (11–14%) protein, (47–53%) fiber, and (10–13.5%) starch [19]. The content of piperine, volatile oil, starch, and fiber can vary markedly in different pepper varieties and is indicative of the quality of black pepper [20]. Black pepper contains about 5–9% of the alkaloids piperine and piperettine and about 1.2–5% of volatile oil [21]. Essential oil is a small portion of a plant material, which consists mainly of terpenes, sequiterpenes, and their derivatives that are responsible for the characteristic aroma, and imparts the identifying flavor and odor most closely associated with the plant itself [22].

Clove is the dried unopened flower bunds of *D. caryophyllus* L. that belong to the Caryophyllaceae family. The dried bulbs resemble a round-headed nail, are dark reddish-brown in color, have a strong aromatic odor and a hot pungent taste. Cloves are now cultivated in many parts of the tropics, particularly Tanzania, Madagascar, Malaysia, India, Srilanka, Jamaica, and French Guiana [23]. Oil of cloves has some antiseptic qualities and is recommended by some dentists as a flavoring aid. Milind and Deepa [24] revealed that clove seeds contains 5.98% protein, 20% total fat, 61.21% carbohydrates, 34.2 fibers, and 5.88% ash. However, it contains also high levels of potassium (1102 mg/100 g) and magnesium (268 mg/100 g). Milind and Deepa [24] also found total saturated fatty acids (5.38%), total monounsaturated fatty acids (1.47%), and total polyunsaturated acids (7.09%).

Although numerous studies on the effects of cultural practices on spices were conducted, changes in their chemical compositions are still far from being finalized specially in Saudi Arabia. Hence, the aim of this study was to investigate the chemical composition, mineral content, and fatty acid profiles of some locally produced spicesand herbs obtained from markets in Saudi Arabia.

## 2. Material and Methods

### 2.1. Materials

Fenugreek, cress, mustard, black cumin, black pepper, and clove were *grown in Saudi Arabia.*

### 2.2. Chemical Composition

Moisture, crude protein, crude fat, crude fiber, and ash were determined according to the AOAC [25] in 2 g, 2 g, 5 g, 2 g, and 5 g sample of each spice. Potassium, magnesium, calcium, zinc, manganese, copper, and iron were extracted with acids from 5 g samples according to McGrath and Cubliffe [26] and their concentrations were detected using a PE model 2380 atomic absorption spectrophotometer.

### 2.3. Fatty Acids Analysis

Oil samples extracted from 5 g seed samples were methylated with 14% boron trifluoride (BF_3_, BDH-Company) in methanol [27]. Analysis of the fatty acids was carried out with a GLC-Varian 6000 gas chromatograph with Flame Ionization Detector (FID), 2 m length, 0.32 cm internal diameter stainless steel column, packed with 15% OV-275, chrome P/AW/80-100 mesh stationary phase which operated at 180°C, injection temperature 230°C, detector temperature 250°C with carrier-gas (Helium) at a flow rate of 25 mL/min, hydrogen flow 30 mL/min, and air flow 300 mL/min. Identification of the fatty acid methyl esters was carried out by comparison of their retention times with that of the standards (Polyscience Corporation, Kit number 61 CX) and the quantities were calculated from the area obtained by the LKB 2200 recorded integrator.

## 3. Statistical Analyses

The factorial experiment in the completely randomized design was done with three replicates. Then all data were statistically analyzed using analysis of variance. 

## 4. Results and Discussion

Chemical composition of spices is given in Table 1. The results indicated that mustard, black cumin, and cress seeds had higher fat content of (38.45%), (31.95%), and (23.19%), respectively, as compared to clove (16.63%), black pepper (5.34%), and fenugreek (4.51%) seeds. It could be noticed that cress, mustard, black cumin, and black pepper had higher protein content that ranged from 20.61% to 25.45%, as compared to fenugreek (12.91%) and clove (6.9%). Crude fiber and ash contents ranged from 6.36 to 23.6% and from 3.57 to 7.1%, respectively. Table 1 shows the obtained results. Kochhar et al. [6] and El Nasri and El Tinay [7] reported that fat, protein, and fiber content of fenugreek seeds ranged from 6.53% to 7.1%, 24.4% to 25.8%, and 6.28% to 9.3%, respectively. On the other hand, Ildikó et al. [12] found that mustard seeds contain 28–32% fat and 28–36% proteins. Gokavi et al. [9], mentioned that cress seeds contain fat, protein and fiber of 27.5, 22.5, and 30%, respectively. It has also been reported that black cumin had 30–40% fat, 20–30% protein, 3.7–4.7% ash, while the protein and fat contents of black pepper ranged from 11–14 and 47–53%, respectively, as reported by Jayashree et al. [19]. Moreover, clove contains 20% fat, 5.98% protein, 34.2% fiber, and 5.88% ash [10].

The low percentage of moisture in cress and black cumin as compared to the others may increase the shelf life of these spices during packaging and storage. They also limit fungal and contamination effects. Data presented in Table 2 shows the mineral contents of the different seeds under investigation. It could be noticed that all seeds contains higher levels of potassium (ranged from 383 to 823 mg/100 g) followed by calcium (ranged from 75 to 270 mg/100 g), magnesium (ranged from 42 to 102 mg/100 g), and iron (ranged from 20.5 to 65 mg/100 g). However, zinc, manganese, and copper metals were found at lower levels. These results differed mainly in the amount of potassium, magnesium, calcium and iron, as compared to previous results obtained by Nergiz and Otles [28], for black cumin and fenugreek seeds (El-Mahdy, and El-Sebaiy [29]). However, Gokavi et al. [9] found that cress seeds contain 1193 mg/100 g potassium. While, clove seeds contain high levels of potassium (1102 mg/100 g) and magnesium (268 mg/100 g) as reported by Milind and Deepa [24]. The differences in the results obtained and that reported in previous studies may be due to environmental factors that prevail in production areas, cultivars used to produce seeds and also due to the different methods used to prepare these local spices.

Fatty acids composition of cress, mustard, black cumin, fenugreek, black pepper, and clove seed oils are presented in Table 3. The major fatty acids in cress and mustard were lenolenic acid (48.43%) and erucic acid (29.81%), respectively. While, linoleic acid was the major fatty acid in black cumin, fenugreek, black pepper, and clove oils being 68.07%, 34.85%, 33.03%, and 44.73%, respectively. Total unsaturated fatty acids were 83.24, 95.62, 86.46, 92.99, 81.34, and 87.82% for cress, mustard, black cumin, fenugreek, black pepper, and clove, respectively. These results are in good agreement with most of the previous studies. Moser et al., [10] reported that oleic (30.60%) and linolenic (29.3%) acids were the major fatty acids in cress seed oil. However, erucic acid (30–40%) was the major fatty acid in mustard oil according to Ali and McKay [11] and Ildikó et al. [12]. Nergiz and Otles [28], Tulukcu [16], and Sultan et al. [30] demonstrated that the predominant fatty acid in black cumin, fenugreek, black pepper and clove seed oils was linoleic acid.

From the results of this study, it could be concluded that the spices and herbs under investigation contain appreciable amounts of nutrients which may serve as beneficial health sources if consumed regularly specially cress and fenugreek and can be used as food supplements for edible oils, besides its uses as a condiments in home. Spices and herbs are used at relatively low levels in foods, these data indicate that spices may provide a meaningful level of protein, fat, and minerals when consumed in a variety of foods. Also, these results were obtained with relatively different results of others and this requires further studies on the impact of soil and weather conditions on the composition of these crops. 

## Figures and Tables

**Table 1:** Chemical composition (%) of cress, mustard, black cumin, fenugreek, black pepper, and clove seeds^∗^.

Analysis	Cress	Mustard	Black cumin	Fenugreek	Black pepper	Clove
Moisture	2.88 ± 0.1	4.36 ± 0.1	2.55 ± 0.2	7.71 ± 0.2	4.68 ± 0.3	7.74 ± 0.2
Crude fat	23.19 ± 0.2	38.45 ± 0.5	31.95 ± 0.3	4.51 ± 0.2	5.34 ± 0.6	16.63 ± 0.3
Crude protein	24.19 ± 0.5	25.39 ± 0.3	20.61 ± 0.3	12.91 ± 0.4	25.45 ± 0.4	6.9 ± 0.4
Crude fiber	11.9 ± 0.4	6.36 ± 0.1	10.37 ± 0.1	13.14 ± 0.3	23.6 ± 0.3	11.47 ± 0.5
Ash	7.1 ± 0.1	4.25 ± 0.1	4.51 ± 0.1	4.23 ± 0.05	3.57 ± 0.1	5.96 ± 0.1
Total carbohydrate^∗∗^	30.74 ± 1.2	21.19 ± 0.9	30.0 ± 1.2	57.5 ± 2.2	37.36 ± 1.4	51.3 ± 2.7

*Means (*n* = 3) ± SD.

^∗∗^Calculated by difference.

**Table 2:** Mineral content (mg/100g) of cress, mustard black cumin, fenugreek, black pepper, and clove seeds^∗^.

Mineral	Cress	Mustard	Black cumin	Fenugreek	Black pepper	Clove
K	663 ± 20.0	383.0 ± 10.0	823.0 ± 30.0	603.0 ± 15.0	663 ± 25.0	650.0 ± 30.0
Mg	102.0 ± 10.0	100.0 ± 8.0	80.0 ± 10.0	42.0 ± 5.0	52.0 ± 8.0	97.0 ± 10.0
Ca	105.0 ± 10.0	159.0 ± 15.0	160.0 ± 10.0	75.0 ± 9.0	195.0 ± 15.0	270.0 ± 20
Zn	3.7 + 0.3	3.4 + 0.3	2.5 ± 0.2	2.4 ± 0.2	0.9 ± 0.1	0.7 + 0.1
Mn	1.1 ± 0.1	1.8 ± 0.2	1.5 ± 0.1	0.9 ± 0.1	3.5 ± 0.2	43.8 ± 1.5
Cu	4.8 ± 0.2	0.7 ± 0.1	0.9 ± 0.1	0.9 ± 0.1	1.3 ± 0.1	0.8 ± 0.1
Fe	21.0 ± 1.1	25.5 ± 1.5	65.0 ± 2.5	25.8 ± 1.2	20.5 ± 0.5	36.0 ± 0.8

*Means (*n* = 3) ± SD.

**Table 3:** Fatty acid (%) extracted from cress, mustard, black cumin, fenugreek, black pepper, and clove seeds^∗^.

Fatty acid	Cress	Mustard	Black cumin	Fenugreek	Black pepper	Clove
C_14 : 0_	1.55	—	1.0	1.38	—	1.29
C_16 : 0_	5.86	3.2	10.5	3.85	3.15	6.21
C_16 : 1_	2.02	—	—	8.29	11.91	20.96
C_18 : 0_	6.56	1.18	2.04	1.78	—	—
C_18 : 1 _(Cis)	15.35	18.32	16.23	8.29	16.17	13.0
C_18 : 1 _(Trans)	4.05	—	—	10.76	9.89	6.20
C_18 : 2_	11.79	23.57	68.07	34.85	33.03	44.73
C_18 : 3_	48.43	23.92	2.16	30.8	10.34	2.93
C_20 : 0_	2.79	—	—	—	15.51	4.68
C_20 : 2_	1.60	—	—	—	—	—
C_22 : 1_	—	29.81	—	—	—	—
TSF	16.76	4.38	13.54	7.01	18.66	12.18
TUSF	83.24	95.62	86.46	92.99	81.34	87.82
